# Vasopressin secretion in sepsis-surviving animals following dehydration

**DOI:** 10.1186/cc13002

**Published:** 2013-11-05

**Authors:** Lucas Favaretto Tazinafo, Tatiana Tocchini Felippotti, Maria José Alves da Rocha

**Affiliations:** 1 Department of Morphology, Physiology and Basic Pathology, Faculty of Dentistry of Ribeirão Preto - USP, Ribeirão Preto, Brazil

## Background

Vasopressin (AVP) plasma levels increase in the early phase of sepsis but remain at basal levels in the late phase of sepsis [1]. It is also known that one-half of septic patients do not properly respond to an osmotic challenge, one of the strongest stimuli for AVP secretion [2]. However, whether these AVP secretion changes persist in sepsis survivors is not known. This study investigated the possible alterations in plasma AVP levels in sepsis-surviving animals.

## Materials and methods

Male Wistar rats were separated into two groups: sepsis induced by cecal ligation and puncture (CLP), or sham animals. They received saline solution (50 mg/ml; s.c) immediately and 12 hours after CLP, and also ceftriaxone (30 mg/kg; s.c.) and clyndamicin (25 mg/kg; s.c.) after every 6 hours for 3 days. Sham animals received the volume of saline corresponding to antibiotic administration. After 10 days, the animals were dehydrated or left as control. After 2 days, the animals were decapitated, and the serum and plasma collected for sodium, hematocrit and hormone determination. The posterior pituitary glands were removed for hormone stock analysis.

## Results

Sepsis-surviving animals presented a higher serum sodium even without the osmotic stimulus (147.8 ± 0.97 SEM vs. 151.4 ± 0.6 SEM mmol/l CLP; *P *< 0.001). Following dehydration, as expected, there was an increase of serum sodium in CLP animals (151.4 ± 0.6 SEM vs. 155.71 ± 0.47 SEM mmol/l; *P *< 0.001) and sham animals (147.8 ± 0.97 SEM vs. 154 ± 0.26 SEM mmol/l dehydrated; *P *< 0.001) with difference between the groups (154 ± 0.26 SEM vs. 155.71 ± 0.47 SEM mmol/l CLP; *P *< 0.041). Hematocrit also increased in both CLP (42.63 ± 1.58 SEM vs. 50.17 ± 1.67% SEM dehydrated; *P *= 0.002) and sham (mean: 41.8 ± 1.43 SEM vs. 49.5 ± 1.0% SEM; *P *= 0.003) groups but without difference between the groups. The animals responded with an increase in the AVP plasma levels (6.12 ± 0.68 SEM vs. 6.16 ± 0.94 SEM pg/ml CLP, *P *> 0.05), and a decrease in AVP neurohypophysis stocks (4.0 ± 1.02 SEM vs. 1.91 ± 0.67 SEM ng/μg CLP; *P *= 0.107), with no difference between the groups.

## Conclusions

The results suggest that sepsis-surviving animals do not present alterations in secretion of AVP in relation to volemia. However, serum sodium results suggest that AVP secretion is impaired in sepsis-surviving animals.

## References

[B1] Correa PB Pancoto JA De Oliveira-Pelegrin GR Carnio EC Rocha MJ Participation of iNOS-derived NO in hypothalamic activation and vasopressin release during polymicrobial sepsis J Neuroimmunol 2007 17 17 25 10.1016/j.jneuroim.2006.10.021 17173980

[B2] Siami S Bailly-Salin J Polito A Porcher R Blanchard A Haymann JP Laborde K Maxime V Boucly C Carlier R Annane D Sharshar T Osmoregulation of vasopressin secretion is altered in the postacute phase of septic shock Crit Care Med 2010 17 1962 1969 20639747 10.1097/CCM.0b013e3181eb9acf

